# Age-Dependent Alterations of Cognition, Mitochondrial Function, and Beta-Amyloid Deposition in a Murine Model of Alzheimer’s Disease—A Longitudinal Study

**DOI:** 10.3389/fnagi.2022.875989

**Published:** 2022-05-02

**Authors:** Martina Reutzel, Rekha Grewal, Aljoscha Joppe, Gunter P. Eckert

**Affiliations:** ^1^Laboratory for Nutrition in Prevention and Therapy, Biomedical Research Center Seltersberg (BFS), Institute of Nutritional Sciences, Justus-Liebig-University Giessen, Giessen, Germany; ^2^Department of Biological Sciences & Cluster of Excellence Macromolecular Complexes, Institute for Molecular Biosciences, Johann Wolfgang Goethe-University Frankfurt, Frankfurt am Main, Germany

**Keywords:** mitochondrial dysfunction, amyloid-beta, Alzheimers’s disease, transgenic mouse models, longitudinal studies

## Abstract

Aging is the main risk factor for sporadic Alzheimer’s disease (AD), which is characterized by the cerebral deposition of β-amyloid peptides (Aβ) and cognitive decline. Mitochondrial dysfunction is also characteristic of the disease and represents a hallmark of both, aging and neurodegeneration. We longitudinally followed Aβ levels, cognition, and mitochondrial function in the same cohort of Thy1-APP_751_SL mice representing a murine model of AD. In the course of time, changes were most prominent at an age of 13 months including the latency time in the passive avoidance test, the activity of complexes I and IV of the mitochondrial respiration chain, and expression of genes related to mitochondrial biogenesis and synaptic plasticity including Peroxisome proliferator-activated receptor gamma coactivator 1-alpha (PGC1-α), CAMP responsive element binding protein 1 (CREB1), and Synaptophysin 1 (SYP1). These changes occurred in parallel with massively increasing cerebral Aβ levels. Other parameters were changed in younger mice including the alteration rate in the Y-maze test and the nesting score when Aβ levels were not changed yet. The results are consistent in the cohort described. However, previous, non-longitudinal studies reported divergent time points for the occurrence of the parameters studied. These findings are discussed in light of the current results.

## Introduction

Alzheimer’s disease (AD) is a neurodegenerative, complex disease. Aging is one major risk factor for sporadic AD. On the molecular level, neurotoxic β-amyloid peptides (Aβ) are deposited in the brains of AD patients after β- and γ-secretase cleavage of the amyloid precursor protein (APP) (Pohland et al., 2018; Hampel et al., 2021). An additional characteristic are the intraneuronal neurofibrillary tangles of hyperphosphorylated tau protein, which leads to dysfunction of synapses and disturbed glucose metabolism in brains of AD patients (Naseri et al., 2019; Zhao and Xu, 2021). It has been shown that a particularly early event in the development of AD and the physiological aging process seems to be the decrease of mitochondrial function. Since mitochondria are the powerhouses of the cell and essential for the synthesis of adenosine triphosphate (ATP) during the oxidative phosphorylation (OXPHOS), mitochondrial dysfunction (MD) is proposed to be an early event in both, the physiological aging process and AD (Hauptmann et al., 2009; Zia et al., 2021). MD is characterized by a decrease in cellular mitochondrial membrane potential (MMP) and ATP levels, a reduced capacity of mitochondrial respiration complexes and impairments during mitochondrial biogenesis. In particular, PGC1-α, a transcription factor responsible for inducing mitochondrial biogenesis, has been shown to be decreased both in AD and during aging. PGC1-α can be activated by AMPK-activated kinase, Sirt1, or creb-1 *via* acetylation or increasing cellular NAD + levels (Li et al., 2017). An increase in PGC1-α was able to reduce the generation of Aβ (Pohland et al., 2018; Mota and Sastre, 2021).

The research of AD and the clarification of exact mechanisms behind the disease is almost inevitable to animal models. Until now, mouse models are most commonly used in AD research. Transgenic AD models are often based on different single or multiple mutations resulting in overexpression of human APP, Aβ, Presenilin 1 or 2 or/and tau proteins (Hall and Roberson, 2012). A variety of promoters are used to overexpress different isoforms of the human amyloid precursor protein. These models are described as a solid basis for visualizing and tracking AD pathology (Hall and Roberson, 2012). However, all models face the major problem that the complexity of AD disease cannot be represented in all its aspects and that they do mainly reflect autosomal dominant rather than sporadic AD. However, they are still an indispensable model for the study of relevant targets and pathways affected in AD. In the current study, mice expressing the human form of APP bearing both, the Swedish (KM670/671NL) and London mutation (V717L) under a murine Thy-1 promoter were used. Pronounced mitochondrial dysfunction in adult Thy1-APP_751_SL mice, already appeared at 3 months (Hauptmann et al., 2009) when elevated intracellular but not extracellular Aβ deposits are present (Blanchard et al., 2003). We recently confirmed forced α- and β-secretase processing of APP leading to enhanced Aβ_1–40_ levels in brains of 3 months old Thy1-APP_751_SL mice and reduced activity of complex IV (C-IV) of the mitochondrial respiration chain (Pohland et al., 2018). In both studies, mitochondrial membrane potential (MMP) and ATP levels were significantly reduced. However, in a more recent study ATP levels were unchanged in brains of 3 months old Thy1-APP_751_SL mice, although C-IV activity and MMP were significantly reduced (Eckert et al., 2020). Previous studies mostly compared only two study time points. To further elucidate the relationship between aging, mitochondrial dysfunction, Aβ formation, and cognition in mice, we initiated a longitudinal study in which we examined the same cohort at different time points. Cognitive performance, Aβ_1–40_ levels, mitochondrial parameters, and mRNA levels of relevant genes were examined at 3, 7, and 13 months of age compared to non-transgenic control mice.

## Materials and Methods

### Animals

#### Thy1-APP_751_SL Mice

Male and female C57Bl/6 mice bearing the human Swedish (S:KM670/671NL) and the London (L:V717I) mutations in the 751 amino acid form of human amyloid-beta precursor protein (AβPP) under control of a murine Thy1 promotor were used. All mice were genotyped by tail biopsies and polymerase chain reaction before and after the experiments as previously described (Pohland et al., 2018). Mice were housed in the institute of pharmacology in Frankfurt a. M. according to the German guidelines for animal care with access to water and food *ad libitum* until they reached the age of 3, 7, and 13 months. Wild-type mice of the same age were used as controls. Experiments were approved by the regional authority (V54-19 c 20/15 – FU/113).

#### Nestbuilding Behavior

Approximately 1 h before the dark phase, the mouse was moved to a single cage if the mouse was previously housed in a group. If the mouse was housed singly, the test could be performed in the “home” cage. All furnishings except litter were removed from the cage and a nestlet was placed in the cage. After 18 h, the test ended. The nests were photographed and scored by two independent observers. Nests were rated from 1 to 5, with 5 representing a perfect nest and 1 representing an unbuilt nest. The evaluation of the nests was analogous to the protocol described in Deacon (2006).

#### Passive Avoidance Test

The test was carried out using a passive avoidance step through system (cat. no. 40533/mice Ugo Basile, Germonio, Italy) and a protocol similar to the protocol published by Shiga et al. (2016). The setup of the passive-avoidance-learning test consisted of two chambers, a light and a dark chamber. The mouse was placed in the light chamber (light intensity 75%). The mouse was acclimated to the light chamber for 30 s before the door opened into the dark chamber. The test was stopped after 180 s or if the mouse entered the dark chamber. In the chamber, the mouse received an electric shock (0.2 mA) for 2 s. After 24 h, the experiment was repeated, with the difference that the acclimation time changed to 5 s and the test was aborted after 300 s. In addition, the electric shock was turned off.

#### One-Trial Y-Maze Test

The mouse was put in one arm of a custom-made Y-shaped maze (material: polyvinyl chloride, length of arms: 36 cm, height of arms: 7 cm, width of arms: 5 cm). Afterward, the mouse was able to explore the maze for 5 min. At the end of the experiment, the number of entries was determined as well as the number of alternations. One alternation was defined as the mouse entered all three arms before it entered an already visited arm. The alternation rate was calculated using the formula [(number of alternations/total number of possible alternations) × 100] (Wolf et al., 2016).

#### Measurement of Soluble Aβ_1–40_

Brain samples were homogenized in 10 times the amount of phosphate-buffered saline containing a protease inhibitor cocktail (Roche cOmplete, Mini Protease Inhibitor Cocktail). Afterward, samples were centrifuged (15,000 × g, 30 min, 4°C) and the supernatants were put into a fresh reaction vessel and stored at −80°C until analysis. Aβ_1–40_ amounts were measured using a specific solid phase sandwich enzyme-linked immunosorbent assay (ELISA; Life Technologies, Carlsbad, CA, United States).

#### Preparation of Dissociated Brain Cells for *ex vivo* Studies

Dissociated Brain Cells (DBC) were freshly prepared using one hemisphere of mouse brain. The method is described in detail in Reutzel et al. (2020). Briefly, the brain was washed in medium 1 (138 mM NaCl, 5.4 mM KCl, 0.17 mM Na_2_HPO_4_, 0.22 mM KH_2_PO_4_, 5.5 mM Glucose × H_2_0, 58.4 mM Sucrose, pH = 7.35), cut into small pieces in 2 ml of medium 1 and was pressed through a 200 μm nylon mesh. Afterward, the brain suspension was filtered through a 102 μm nylon mesh. The cell homogenate was centrifuged (2,000 rpm, 5 min, 4°C) and the resulting pellet was dissolved in 20 ml medium 2 (110 mM NaCl, 5.3 mM KCl, 1.8 mM CaCl_2_ × 2 137 H20, 1 mM MgCl_2_ × 6 H20, 25 mM Glucose × H20, 70 mM Sucrose, 20 mM HEPES). The centrifugation step was repeated twice and the pellet dissolved in 4.5 ml Dulbecco’s Modified Eagle Medium (DMEM) without supplements. For ATP measurements twelve replicates of 50 μl cell suspension were seeded into a 96 well plate. Respectively 6 wells were incubated with sodium nitroprusside (0.5 mM). For determination of the mitochondrial membrane potential (MMP) 250 μl were seeded into 24 well plate and 6 wells were incubated with 2 mM sodium nitroprusside. Afterward, the plates were incubated for 3 h in a humified incubator (5% CO_2_) before measurement of mitochondrial membrane potential or ATP. The remaining suspension was stored for protein determination at −80°C).

#### Measurement of Mitochondrial Membrane Potential

For the measurement of mitochondrial membrane potential (MMP) DBC’s were incubated for 15 min in a humified incubator (5% CO_2_) with 0.4 μM of the fluorescence dye Rhodamine-123. The reaction was stopped by adding 250 μl of Hank’s balanced salt solution (HBSS) into each well. The plate was centrifuged (914 g, 5 min, room temperature), the medium aspirated and new HBSS was added into the wells. DBC were triturated and MMP was measured by reading the R123 fluorescence at an excitation wavelength of 490 nm and an emission wavelength of 535 nm Victor X3 multilabel counter). The measurement was repeated four times and the values normalized to protein content of the sample.

#### Measurement of Adenosine Triphosphate Levels

Adenosine Triphosphate Levels (ATP) concentrations were determined in DBC’s with the ViaLight Plus bioluminescence kit (Lonza, Walkersville, MD, United States). After the incubation, the plate was removed from the incubator and allowed to cool to room temperature for 10 min. All wells were incubated for 10 min with 25 μl lysis buffer in the dark. Next, wells were incubated with 50 μl monitoring reagent. The measurement was conducted according to the manufactures instructions. The emitted light (bioluminescence) was recorded using a Victor X3 multilabel counter and is linearly related to ATP content.

#### Preparation of Isolated Brain Mitochondria

Half a brain hemisphere (the frontal part) was used for the isolation of brain mitochondria for mitochondrial respiration as previously described (Hagl et al., 2013). Briefly, the sample was homogenized using a potter equipped with a Teflon^®^ pistil in 2 ml mitochondrial respiration medium (MiR05) containing a protease inhibitor cocktail (PI, complete; Roche, Mannheim, Germany). Afterward, the homogenate was centrifuged (1,400 g, 7 min, 4°C). A second centrifugation was conducted with the supernatant for 3 min followed by a centrifugation at 100,000 g for 5 min at 4°C. The resulting pellet containing the mitochondria was dissolved in 250 μl MIRO5 + PI and 80 μl of the solution was used for the measurement of mitochondrial respiration using an Oxygrapph-2k respirometer (Oroboros, Innsbruck, Austria). The remaining mitochondria solution was frozen in liquid nitrogen for citrate synthase activity (120 μl) and protein determination (50 μl).

#### High-Resolution Respirometry

After injecting 80 μl of the mitochondria suspension into the Oxygraph-2k chamber a protocol elaborated by Prof. Dr. Erich Gnaiger was used including several inhibitors, uncouplers and substrates of the respiratory chain system (Krumschnabel et al., 2015). A detailed description of the different steps of the protocol is described in Hagl et al. (2016). Briefly, the capacity of the oxidative phosphorylation (OXPHOS) was determined using complex-I related substrates pyruvate (5 mM) and malate (2 mM) and ADP (2 mM) followed by the addition of succinate (10 mM). Mitochondrial integrity was measured by addition of cytochrome c (10 μM). Oligomycin (2 μg/ml) was added to determine leak respiration [leak (omy)] and afterward uncoupling was achieved by carbonyl cyanide p-(trifluoromethoxy) phenyl-hydrazone (FCCP, injected stepwise up to 1–1.5 μM). Complex II respiration was measured after the addition of rotenone (0.5 μM). Complex III inhibition was achieved by the addition of antimycin A (2.5 μM) and was subtracted from all respiratory parameters. COX activity was measured after ROX determination by applying 0.5 mM tetramethylphenylenediamine (TMPD) as an artificial substrate of complex IV and 2 mM ascorbate to keep TMPD in the reduced state. Autoxidation rate was determined after the addition of sodium azide (>100 mM), and COX respiration was additionally corrected for autoxidation.

#### Transcription Analysis by Quantitative Real-Time PCR

RNA isolation was conducted with the RNeasy Mini Kit (Qiagen, Hilden, Germany) according to the manufacturer’s instructions using ∼ 20 mg RNAlater stabilized brain tissue samples (Qiagen, Hilden, Germany). RNA was quantified measuring the absorbance at 260 and 280 nm using NanoDrop™ 2000c spectrometer (Thermo Fisher Scientific, Waltham, MA, United States). Complementary DNA was synthesized from 250 ng total RNA using the iScript cDNA Synthesis Kit (BioRad, Munich, Germany) according to the manufactuer’s instructions and was stored at −80°C. qRT-PCR was conducted using a CfX 96 Connect™ system (BioRad, Munich Germany) (Reutzel et al., 2020). All primers were received from biomol (Hamburg, Germany) or biomers (Ulm, Germany). cDNA for qRT-PCR was diluted 1:5 with RNase free water (Qiagen, Hilden, Germany) and all samples were performed in triplicates. PCR cycling conditions were an initial denaturation at 95°C for 3 min, followed by 45 cycles of 95°C for 10 s, 58°C for 45 s and 72°C for 29 s. Gene expression was analyzed using the –(2ΔΔ C_*q*_) method using BioRad CfX manager software and were normalized to the expression levels of beta 2 microglobulin (B2M) and phosphoglycerate kinase 1 (PGK1). All primer sequences, product sizes and primer concentrations used for quantitative real-time PCR (QRT-PCR) are listed in Table 1.

**TABLE 1:** Oligonucleotide primer sequences, product sizes and primer concentrations for quantitative real-time pcr; bp: base pair.

Primer	Sequence	Product size (bp)	Concentration (μM)
AMP-activated protein kinase (β-subunit) (β-AMPK)	5′-agtatcacggtggttgctgt-3′ 5′-caaatactgtgcctgcctct-3′	190	0.1
Beta-2-Microglobulin (B2M)	5′-ggcctgtatgctatccagaa-3′ 5′-gaaagaccagtccttgctga-3′	198	0.4
Brain-derived neurotrophic factor (BDNF)	5′-gatgccagttgctttgtctt-3′ 5′-atgtgagaagttcggctttg-3′	137	0.1
CAMP responsive element binding protein 1 (CREB1)	5′-tagctgtgacttggcattca-3′ 5′-ttgttctgtttgggacctgt-3′	184	0.5
Citrate synthase (CS)	5′-aacaagccagacattgatgc-3′ 5′-atgaggtcctgctttgtcct-3′	184	0.1
Complex I (CI)	5′acctgtaaggaccgagaga-3′ 5′-gcaccacaaacacatcaaaa-3′	227	0.1
Complex IV (CIV)	5′-ctgttccattcgctgctatt-3′ 5′-gcgaacagcactagcaaaat-3′	217	0.1
Growth-associated protein (GAP43)	5′agggagatggctctgctact-3′ 5′gaggacggggagttatcagt-3′	190	0.15
Mitochondrial transcription factor A (TFAM)	5′-agccaggtccagctcactaa-3′ 5′-aaacccaagaaagcatgtgg-3′	166	0.5
Nuclear respiratory factor 1 (NRF-1)	5′-tcggagcacttactggagtc-3′ 5′-ctagaaaacgctgccatgat-3′	228	0.5
Peroxisome proliferator-activated receptor gamma coactivator 1-alpha (PGC1-α)	5′-tgtcaccaccgaaatcct-3′ 5′-cctggggaccttgatctt-3′	124	0.05
Phosphoglycerate kinase-1 (PGK1)	5′-gcagattgtttggaatggtc-3′ 5′-tgctcacatggctgacttta-3′	185	0.4
Sirtuin-1 (Sirt-1)	5′-gtgagaaaatgctggcctaa-3′ 5′-ctgccacaggaactagagga-3′	161	1
Synaptophysin 1 (SYP1)	5′-tttgtggttgttgagttcct-3′ 5′-gcatttcctccccaaagtat-3′	204	0.1

#### Citrate Synthase Activity

Citrate synthase (CS) activity in isolated mitochondria was determined photometrically as previously published (Pohland et al., 2018; Reutzel et al., 2020). Briefly, a sample of the isolated mitochondria in MiR05 was thawed on ice and the reaction medium [100 μl of 0.1 mM 5,5’-dithiobis-(2-nitrobenzoic acid) (DTNB), 25 μl of 10% Triton X-100, 50 μl of 10 mM oxaloacetate, 25 μl of 12.2 mM acetyl coenzyme A, and 790 μl purified water] was mixed and warmed to 30°C for 5 min. Afterward, 10 μl isolated mitochondria were added to the reaction mixture and transferred into a 10 mm quartz cuvette (Hellma^®^ Analytics, Müllheim, Germany). CS activity was measured at 412 nm using a GENESYS 5 spectrophotometer (Spectronic via Thermo Fisher Scientific, Waltham, MA, United States). All samples were measured in duplicates.

#### Protein Quantification

Protein content was measured with the Pierce™ Protein Assay Kit (Thermo Fisher Scientific, Waltham, MA, United States) according to the Manufacturer’s instructions.

#### Statistical Analysis

Unless otherwise stated, values are presented as mean ± standard error of the mean (SEM). Statistical analyses were performed by applying a two sided, unpaired Student’s *t*-test and for multiple comparisons a one-way ANOVA with Tukey’s multiple comparison post test (Prism 8.0 GraphPad Software, San Diego, CA, United States). Statistical significance was defined for p*-values of <0.05, p** < 0.01, p*** < 0.001 and p**** < 0.0001.

## Results

To assess the relationship between Aβ formation, cognitive performance and mitochondrial brain function, we used an amyloid-based mouse strain and exclusively followed these parameters longitudinally from one cohort over 13 months. Thy1-APP_751_SL mice were examined at the ages of 3, 7, and 13 months in comparison to their non-transgenic controls.

### Measurement of Soluble Amyloid-β_1–40_ Brain Levels

**FIGURE 1:**
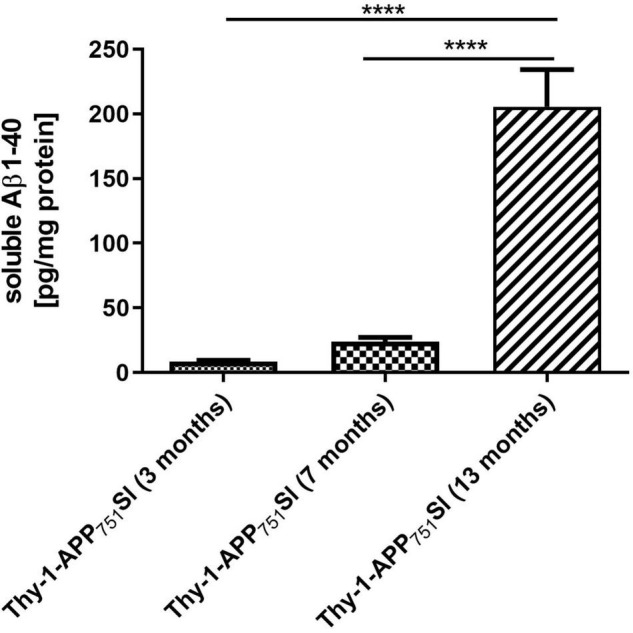
Soluble human Aβ_1–40_ levels in homogenates isolated from brains of 3, 7, and 13 months old Thy1-APP_751_SL mice determined using ELISA. Data represent means ± SEM. *n* = 10; One-Way ANOVA with Tukey’s *post-hoc* test with ****p < 0.0001.

Thy1-APP_751_SL mice aged 13 months showed significantly increased levels of soluble human Amyloid β_1–40_ brain levels (206 pg/mg protein) compared to mice aged 7 (24 pg/mg protein) and 3 months (9 pg/mg protein) (Figure 1).

### Effect on Cognition and Nest-Building Behavior During Aging

**FIGURE 2:**
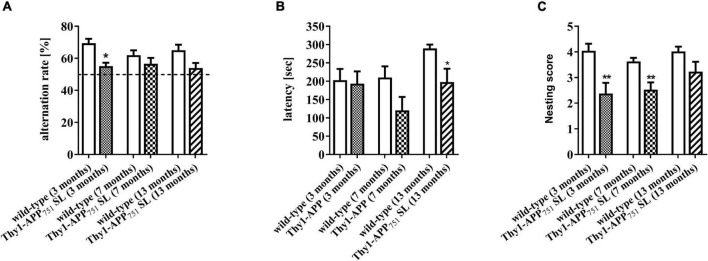
Alternation rate in the Y-Maze spontaneous alternation test **(A)**, latency time day 2 in the Passive Avoidance test **(B)**, and nesting scores **(C)** of 3, 7, and 13 months old Thy1-APP_751_SL mice compared to wild-type mice of the same age. *n* = 13 *t*-test against wild-type mice of the same age with *p < 0.05 and **p < 0.01.

Thy1-APP_751_SL mice aged 3, 7, and 13 months were tested over a time period of 5 minutes in the one-trial-Y-Maze test compared to wild-type mice of the same age. The Y-Maze test provides insight into motor skills and spatial learning memory (Wolf et al., 2016). Thy1-APP_751_SL aged 3 months showed a significant reduced alternation rate compared to control animals of the same age (*p* < 0.05). At ages 7 and 13 months, there was a numerical decrease in the ratio of alternation compared with controls of the same age (Figure 2A). The results suggest early impairments of the spatial learning memory in 3 months old Thy1-APP_751_SL mice.

Thy1-APP_751_SL mice at the age of 13 months showed a significant reduced latency time in the Passive Avoidance test compared to wild-type mice (Figure 2B). The passive avoidance test gives information on how a mice can remember a negative stimulus and may be an indicator for the function of the long-term memory (Shiga et al., 2016). Results indicate deficits in the long-term memory of 13 months old Thy-1-APP_751_ mice. At the ages of 3 and 7 months, Thy1-APP_751_SL mice had a significant deficit in their nest building behavior compared to wild-type mice (Figure 2C). This test is used to assess thermoregulatory behavior, positive motivational states and welfare in mice (Deacon, 2006; Gaskill et al., 2013). Results may indicate decreased motivated behavior and increased stress levels of those mice.

### Effects on Mitochondrial Bioenergetics During Aging in Thy1-APP_751_SL Mice

The mitochondrial respiratory chain in the inner mitochondrial membrane is the main site of ATP formation. The respiratory chain builds up a proton gradient *via* a total of four respiratory chain complexes [complex I, NADH: ubiquinone oxidoreductase (CI); complex II, succinate-coenzyme Q reductase (CII); complex III, cytochrome-c oxidoreductase (CIII); complex IV, cytochrome-c oxidase (CIV)] the so-called membrane potential, which is the driving force for ATP synthase [F_1_/F_0_-ATPase (CV)] for the synthesis of ATP from ADP and inorganic phosphate (Larosa and Remacle, 2018).

**FIGURE 3:**
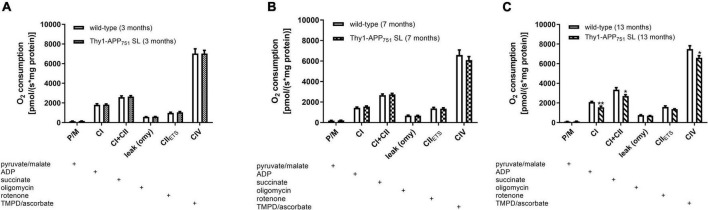
Mitochondrial Respiration in isolated brain mitochondria from 3 **(A)**, 7 **(B)**, and 13 **(C)** months old Thy1-APP_751_SL mice compared to wild-type control animals of the same age; the addition of a substance into the oxygraph chamber is indicated with a plus sign; *n* = 10; mean ± SEM and *t*-test against wild-type mice of the same age with *p < 0.05 and **p < 0.01.

Additionally, mitochondrial respiration was measured in isolated brain mitochondria (Figures 3A–C). Thy1-APP_751_SL mice at the age of 13 months showed significant deficits in CI, CI + CII, and CIV respiration compared to wild-type mice of the same age. However, no significant changes in citrate synthase activity, a mitochondrial mass marker (Rabøl et al., 2010), were observed at any measurement time point (data not shown). MMP-levels showed a numerically increase at the age of 13 months (Table 2). ATP- levels in brains of 3, 7, and 13 months old Thy1-APP_751_SL were measured compared to wild-type mice of same age. Basal ATP-levels were significant elevated in brains of 7 months old Thy1-APP_751_SL mice (*p* < 0.05).

**TABLE 2:** Basal ATP- and MMP-levels in dissociated brain cells of 3, 7, and 13 months old Thy1-APP_751_SL mice compared to wild-type (wt) control animals of the same age.

	wt control	Thy1-APP_751_SL	wt control	Thy1-APP_751_SL
Age (months)	ATP (nmol/mg protein)	ATP (nmol/mg protein)	Fluorescence (AU/mg protein)	Fluorescence (AU/mg protein)
3	1.0 ± 0.1	1.2 ± 0.1	77546 ± 5287	86135 ± 6912
7	1.1 ± 0.1	1.6 ± 0.2*	123255 ± 8162	129606 ± 12008
13	1.2 ± 0.1	1.6 ± 0.2	115747 ± 20668	130098 ± 14436

n = 9; mean ± SEM and t-test against wild-type mice of the same age with *p < 0.05.

### Effect on Gene Expression During Aging

Expression of genes involved in mitochondrial biogenesis and mitochondrial respiration, as well as genes which are involved in synaptic plasticity and neurogenesis were measured in brains of 3, 7, and 13 months old Thy1-APP_751_SL mice compared to wild-type mice of the same age. Expression of β-AMPK and Mitochondrial transcription factor A (TFAM) were significant reduced starting at an age of 7 months indicating impaired mitochondrial biogenesis. All other genes involved in the formation of new mitochondria (CREB1, NRF1, PGC1-α, and SIRT-1) showed a significant decrease at the age of 13 months compared to wild-type mice of the same age. Genes involved in synaptic plasticity, learning behavior and neuronal remodelling (GAP43, SYP1, and BDNF) showed a significant decline in 13 months old Thy1-APP_751_SL mice compared to wild-type animals. The mRNA expression of complex IV of the respiratory chain showed an increase in brains of 3 months old transgenic mice. As age progresses, mRNA expression of complex I and IV drops significantly at the age 13 months in Thy1-APP_751_SL (Table 3). Results of mRNA expression patterns of wild-type mice during aging are shown in the supplements.

**TABLE 3:** Relative normalized mRNA Expression of 3, 7, and 13 months old Thy1-APP_751_SL mice compared to wild-type mice of the same age.

Gene	Thy1-APP_751_SL (3 months)	Thy1-APP_751_SL (7 months)	Thy1-APP_751_SL (13 months)
AMP-activated protein kinase (β-AMPK)	116.6 ± 9.2	87.9 ± 3.7*	72.7 ± 2.3*
Brain-derived neurotrophic factor (BDNF)	119.3 ± 27.8	98.6 ± 18.3	49.8 ± 6.8*
CAMP responsive element binding protein 1 (CREB1)	114.1 ± 5.5	96.0 ± 6.5	68.1 ± 4.3**
Citrate synthase (CS)	107.9 ± 9.5	94.8 ± 4.1	64.4 ± 3.8****
Complex I (CI)	117.1 ± 7.5	95.55 ± 6.4	64.1 ± 4.2****
Complex IV (CIV)	126.0 ± 6.3**	91.7 ± 7.1	60.5 ± 6.3****
Growth-associated protein (GAP43)	120.8 ± 8.8	96.5 ± 9.1	70.8 ± 6.5**
Mitochondrial transcription factor A (TFAM)	113.3 ± 6.5	89.2 ± 4.6*	67.9 ± 4.5****
Nuclear respiratory factor 1 (NRF-1)	100 ± 4.1	98.5 ± 6.6	65.9 ± 3.8****
Peroxisome proliferator-activated receptor gamma coactivator 1-alpha (PGC1-α)	96.1 ± 6.7	91.1 ± 5.7	63.5 ± 6.4**
Sirtuin-1 (Sirt-1)	109.0 ± 6.5	93.8 ± 5.1	71.2 ± 4.6***
Synaptophysin 1 (SYP1)	111.5 ± 8.5	94.9 ± 6.3	71.8 ± 4.5**

Wild-type mice of the same age are defined as 100%. Results are normalized to the expression levels of B2M and PGK1. n = 9; t-test against wild-type mice of the corresponding age with *p < 0.05; **p < 0.01; ***p < 0.001; ****p < 0.0001.

## Discussion

As Alzheimer’s disease (AD) is the most prevalent neurodegenerative disease of aging, we followed the longitudinal brain aging process in Thy1-APP_751_SL mice as an model of early AD (Hauptmann et al., 2009). Additionally, on the molecular level, it appears that there is a decline in mitochondrial function in both, the aging process and AD (Cadonic et al., 2016; Perez Ortiz and Swerdlow, 2019). Research into AD makes it essential to work with animal models that reflect as many characteristics of this multifactorial disease as possible. However, no animal model exists that can represent all of the extensive molecular changes as well as cognitive impairments in its full extent (Hall and Roberson, 2012; Puzzo et al., 2015). We therefore examined our Aβ-based mouse model, which has not been studied longitudinally before, over a period of 13 months. Neuron-specific expression of the human APP gene depends on the promoter used. While the platelet-derived growth factor B-chain (PDGF-B) and thymocyte differentiation antigen 1 (THY-1) promoters are very specific in leading to APP overexpression in neurons, the prion protein (PrP) promoter is much more active than the first two, but less specific and leads to increased APP expression in liver, kidney, and other tissues (Asante et al., 2002). Cognitive deficits measured by the Y-Maze, passive avoidance, and nesting tests were observed as early as 3 months of age. In the current study, most biochemical changes, including increased Aβ_1–40_ levels, manifested at 13 months of age in the brains of Thy1-APP_751_SL mice.

### Cognitive Function and Aβ_1–40_ Levels

Previous studies reported elevated levels of human Aβ starting at an age of 3 months in brains of Thy1-APP_751_SL mice (Blanchard et al., 2003; Hauptmann et al., 2009; Pohland et al., 2018; Eckert et al., 2020). In the current study soluble Aβ_1–40_ levels were relatively low at the age of 3 months (9 pg/mg protein), at age of 7 months, only 24 pg/mg soluble Aβ_1–40_ were determined. At the age of 13 months, significantly elevated Aβ_1–40_ levels of 206 pg/mg protein were formed in the brain of Thy1-APP_751_SL mice. These results are in contrast to previous studies including our owns (see Table 4), in which almost three times higher Aβ values were reported at 3 months of age in the same mouse strain (Schuessel et al., 2005; Pohland et al., 2018; Eckert et al., 2020). We can only speculate about the reasons, which may lie in the housing conditions or in the handling of the mice which, however, have not deviated from our standard protocols. In any case, the overall results of the cohort appear to be consistent over time. The most pronounced changes in biochemical parameters were associated with the highest Aβ levels in the oldest mice (see below). Interestingly, cognitive functions were already altered in younger mice of the same cohort.

**TABLE 4:** Results of studies that determined mitochondrial function in Thy1-APP_751_SL mice compared with the current longitudinal study focusing on amyloid levels in the brain; n.d. not determined.

Study	Age [months]	Human Aβ40 [pg/mg]	Mitochondrial function	Cognitive functions
Current study	3, 7, 13	9 (3 months) 24 (7 months) 206 (13 months)	ATP ↑ (7 months) MMP ↔ C-IV ↓ (13 months)	Deficits starting at the age of 3 months
Eckert et al., 2020	3	∼ 60	ATP ↔ MMP ↓ C-IV ↓ (3 months)	n.d.
Eckert et al., 2008	3	n.d.	ATP ↓ MMP ↓	n.d.
Hauptmann et al., 2009	3, 6	n.d.	ATP ↓ (3, 6 months) MMP ↓ (3, 6 months)	n.d.
Keil et al., 2004	3	n.d.	ATP ↓ MMP ↓	n.d.
Pohland et al., 2018	3	∼ 60	ATP ↓ MMP ↓ C-IV ↓ (3 months)	n.d.
Schuessel et al., 2005	3, 12	∼100 (3 months)	n.d.	n.d.
		∼150 (12 months)	Increased oxidative damage	

*Unless otherwise stated wild-type animals of the same age served as control. Arrows indicate when significant effects were observed (↑ increase, ↓ decrease, ↔ no significant effect).*

Cognitive functions and behavior were assessed using three different behavioral tests. Deficits in nest-building behavior and spatial learning memory of transgenic animals were already observed at the age of 3 months. Deterioration in long-term memory was noted at 7 months of age and manifested at 13 months of age. Most studies in transgenic AD mouse models suggesting cognitive dysfunction due to increasing soluble Aβ levels as well as Aβ plaque deposition (Huber et al., 2018; He et al., 2020; Johnson et al., 2020). Since cerebral Aβ levels of 7-months old animals were very low, one could conclude that the onset of cognitive dysfunction may not be directly related to elevated APP processing. Accordingly, for 18-month-old APP/PS1 mice, Vartak et al. (2019) did not find a correlation between cognitive function and soluble/insoluble Aβ_1–40_ and Aβ_1–42_ content in the brain. Eckert et al. (2008) observed a marked increase in soluble intracellular Aβ levels starting at 3 months of age in Thy1-APP_751_SL mice and a parallel correlation to reduced mitochondrial function, although cognitive functions were not determined in this study.

### Longitudinal Mitochondrial Parameters in Thy1-APP_751_SL Mice

In agreement with previous studies, a significantly reduced activity of mitochondrial C-IV activity was measured (Table 4). However, this deficit again did not occur until 13 months of age, at relatively high Aβ levels of 203 pg/mg protein. Previous studies reported changes in C-IV activity as early as 3 months of age, when Aβ levels were around 60 pg/mg protein (Pohland et al., 2018; Eckert et al., 2020). At 7 months of age, our animals showed only a small and non-significant decrease in C-IV activity. However, at this time point, ATP levels were significantly increased, possibly as a result of a compensatory response.

Interestingly, the improvement in C-IV activity and mitochondrial membrane potential in 3-month-old Thy1-APP_751_SL mice treated with olesoxime was accompanied by an increase in Aβ levels (Eckert et al., 2020). In this study, the ATP levels in the brains of the 3 month old animals, as in the present one, did not show any change.

### Longitudinal Gene Expression Patterns in Thy1-APP_751_SL Mice

Again, the changes in the brains of Thy1-APP_751_SL mice were greatest at 13 months of age. The expression levels of all genes studied were significantly reduced at this age. In addition to the plasticity markers SYP1 and GAP43, this also affected all mitochondria-related genes. In 7 months old Thy1-APP_751_SL mice there is a decrease in the mitochondrial transcription factor A (TFAM) and AMPK-activated protein kinase (β-AMPK), which is one of the key activator for mitochondrial transcription (Li et al., 2017). In accordance with the literature, it is described that in 3xTG-AD mice the mRNA expression of TFAM and other genes involved in mitochondrial biogenesis seem to be already decreased from 1 month of age. This observation was made before a significant deposition of Aβ oligomers took place in brains of transgenic mice (Singulani et al., 2020). Furthermore, the literature describes that in 12-month-old APP mice and hippocampal neurons significant deficits in mitochondrial biogenesis and mitochondrial dynamics take place, which are linked to deficits in the cognitive function of the mice (Manczak et al., 2018). Additionally, in hippocampal neurons Aβ_25–35_ incubation for 24 h leads to a significant decrease in AMPK and SIRT1 expression (Dong et al., 2016). New mitochondria formation, which is indispensable for maintaining energy supply, is significantly reduced in brains of AD patients, as well as in cellular models of AD. For example, Sheng et al. detected reduced mRNA and protein levels of PGC1-α, NRF-1, NRF2-2, and TFAM in postmortem AD brains and cellular models of AD (Sheng et al., 2012). Directly relevant to our results reduced levels of genes involved in mitochondrial biogenesis were determined in brains of 6- and 12 months old APP transgenic mice (Manczak et al., 2016, 2018; Song et al., 2018).

### Limitations

There are limitations to our study. Based on our previous findings, we aimed to investigate the deterioration of mitochondrial and cognitive function with increasing age. However, contrary to expectations, the parameters showed changes only at older ages. Although the cause of this phenomenon cannot be substantiated with evidence, the data nevertheless show that mitochondrial parameters and cognition deteriorate with elevated Aβ levels with increasing age.

## Conclusion

A strength of our investigation is the observation of a cohort of Thy1-APP_751_SL mice over a relatively long period of time. The data suggest a strong relationship between the Aβ formation in the brain of the animals and the measured parameters. This underlines the importance of aging processes for a possible therapy or prevention of the disease.

## Data Availability Statement

The original contributions presented in the study are included in the article/Supplementary Material, further inquiries can be directed to the corresponding author.

## Ethics Statement

The animal study was reviewed and approved by V54-19 c 20/15 – FU/113.

## Author Contributions

MR generated the figures and involved in most of the experiments during the study and guided AJ. AJ was responsible for the measurement of ELISA and qPCR. MR, RG, and AJ were involved in the animal studies and measured mitochondrial function. GE supervised the work. MR and GE wrote the manuscript. All authors read and approved the final manuscript.

## Conflict of Interest

The authors declare that the research was conducted in the absence of any commercial or financial relationships that could be construed as a potential conflict of interest.

## Publisher’s Note

All claims expressed in this article are solely those of the authors and do not necessarily represent those of their affiliated organizations, or those of the publisher, the editors and the reviewers. Any product that may be evaluated in this article, or claim that may be made by its manufacturer, is not guaranteed or endorsed by the publisher.
