# Supplementary material for: Age-Dependent Alterations of Cognition, Mitochondrial Function, and Beta-Amyloid Deposition in a Murine Model of Alzheimer’s Disease—A Longitudinal Study
Source: Front Aging Neurosci. 2022 May 2;14:875989. doi: 10.3389/fnagi.2022.875989 (PMC9108248; doi:10.3389/fnagi.2022.875989)
Supplement: Supplementary file 1 [file Table_1.DOCX]

**Supp.1**: Relative normalized mRNA Expression of 7 and 13 months old wild-type mice compared to mice aged 3 months. 3 months old animals are defined as 100 %. Results are normalized to the expression levels of B2M and PGK1. n= 10; One-way ANOVA with *p < 0.05; **p < 0.01; ***p < 0.001; ****p < 0.0001. # indicates one-way ANOVA compared to 7 months old animals with ^#^p < 0.05 and ^##^p < 0.01.

| **Gene** | **Wild-type [7 months]** | **Wild-type [13 months]** |
| --- | --- | --- |
| AMP-activated protein kinase (β-AMPK) | 92.4 ± 3.6 | 86.9 ± 5.8 |
| Brain-derived neurotrophic factor (BDNF) | 38.9 ± 3.7* | 25.1 ± 3.3** |
| CAMP responsive element binding protein 1 (CREB1) | 90.3 ± 3.3 | 89.2 ± 5.9 |
| Citrate synthase (CS) | 102.7 ± 4.6 | 95.1 ± 2.4 |
| Complex I (CI) | 109.8 ± 3.2 | 93.9 ± 3.3 |
| Complex IV (CIV) | 123.1 ± 4.7** | 111.3 ± 4.8 |
| Growth-associated protein (GAP43) | 136.4 ± 7.4** | 113.9 ± 6.6 |
| Mitochondrial transcription factor A (TFAM) | 106.3 ± 2.3 | 102.1 ± 3.7 |
| Nuclear respiratory factor 1 (NRF-1) | 111.4 ± 8.0 | 110.0 ± 4.3 |
| Peroxisome proliferator-activated receptor gamma coactivator 1-alpha (PGC1-α) | 99.4 ± 5.4 | 118.7 ± 4.5*^#^ |
| Sirtuin-1 (Sirt-1) | 122.8 ± 6.5* | 99.1 ± 3.7^#^ |
| Synaptophysin 1 (SYP1) | 128.5 ± 4.8 | 175.0 ± 12.0****## |
